# Implication of the Global Initiative for Chronic Obstructive Lung Disease 2023 report for resource-limited settings: tracing the G in the GOLD

**DOI:** 10.1183/13993003.00484-2023

**Published:** 2023-06-15

**Authors:** Bruce J. Kirenga, Patricia Alupo, Frederik van Gemert, Rupert Jones

**Affiliations:** 1 Makerere University Lung Institute, Kampala, Uganda; 2 Department of Medicine, Makerere University, Kampala, Uganda; 3 Department of Health Sciences, Groningen Research Institute for Asthma and COPD, University Medical Center Groningen, Groningen, The Netherlands; 4 Research and Knowledge Exchange, Plymouth Marjon University, Plymouth, UK

## Abstract

The new Global Initiative for Chronic Obstructive Lung Disease (GOLD) 2023 report provides a very useful synthesis of available scientific evidence to guide COPD management, research and prevention, as always [1, 2]. Important changes include the revision of the definition of the condition and the replacement of groups C and D with E, which highlights the importance of exacerbations in COPD [2].

## Abstract 2

**COPD guidelines still have blind spots for low-resource settings. If GOLD is truly to be considered a global report on COPD, more attention will need to be paid to practical solutions in global settings.** https://bit.ly/3zusjOd

*To the Editor*:

The new Global Initiative for Chronic Obstructive Lung Disease (GOLD) 2023 report provides a very useful synthesis of available scientific evidence to guide COPD management, research and prevention, as always [1, 2]. Important changes include the revision of the definition of the condition and the replacement of groups C and D with E, which highlights the importance of exacerbations in COPD [2].

We reviewed the report to examine how well it addresses the issues surrounding COPD in Africa and other resource-limited settings. We commend the report for highlighting that COPD not related to cigarette smoking constitutes half of all COPD globally. In resource-limited settings, exposure to biomass smoke while cooking, outdoor air pollution and occupational exposure are the biggest drivers of COPD, especially among women. Pulmonary tuberculosis and HIV are also notable risk factors for COPD in this setting, with over 20% of COPD patients reporting having had a history of pulmonary tuberculosis [3].

In a study in rural Uganda, only 8% of women with COPD were current smokers, 84% had never smoked and >90% of people in the cohort had biomass exposure throughout their lives [4]. The report could have included more details on the importance of reducing or eliminating household air pollution exposure. Furthermore, the report points out the role of poverty-related exposures as another significant contributor to COPD in resource-limited settings. There are, however, other poverty-related factors associated with COPD in these settings, such as nutrition and infection, especially in early childhood. The report identifies childhood factors that impair lung growth and result in COPD in adulthood. Identified factors include prematurity, low birth weight, maternal smoking during pregnancy, repeated respiratory infections and poor nutrition, among others. We believe that household air pollution should be mentioned here as well. Young children (even before birth when a pregnant woman is cooking) are exposed to high levels of household air pollution, probably causing lifelong harm.

The report states that spirometry is needed to confirm the diagnosis of COPD, but spirometry is rarely available in Africa [5]. Given the prevalence of COPD, resources need to be made available to make the diagnosis. Screening questionnaires or peak expiratory flow testing are better than nothing. Our group used symptom and risk factor screening followed by peak expiratory flow testing and found it to be fairly accurate and feasible [6].

The report now recommends a combination of long-acting β-agonist (LABA) and long-acting muscarinic antagonist (LAMA) for all patients in groups B and E, with a possibility of adding inhaled corticosteroids to those with high peripheral blood eosinophils. Generally, inhaled medications are hard to access in most of Africa [7]. LAMA/LABAs are not widely available. Cheaper and more available alternatives should be included as alternatives in low-resource settings. However, other affordable interventions should be prominently promoted in resource-limited settings, including vaccinations for respiratory infections, pulmonary rehabilitation and surgical treatments.

In conclusion, the report is an excellent resource for understanding COPD and how best to manage it now. However, we agree with Tabyshova *et al.* [8] that COPD guidelines still have blind spots for low-resource settings. This matters, given most of the morbidity and more than 90% of COPD deaths occur in low-resource settings [9]. If this is truly to be considered a global report on COPD, more attention will need to be paid to practical solutions in global settings.

## Shareable PDF

10.1183/13993003.00484-2023.Shareable1 This one-page PDF can be shared freely online.
Shareable PDF ERJ-00484-2023.Shareable
