# Supplementary material for: Implication of the Global Initiative for Chronic Obstructive Lung Disease 2023 report for resource-limited settings: tracing the G in the GOLD
Source: Eur Respir J. 2023 Jun 15;61(6):2300484. doi: 10.1183/13993003.00484-2023 (PMC10269374; doi:10.1183/13993003.00484-2023)
Supplement: Supplementary file 1 [file ERJ-00484-2023.Shareable.pdf]

EUROPEAN RESPIRATORY JOURNAL
CORRESPONDENCE
B.J. KIRENGA ET AL.

Implication of the Global Initiative for Chronic Obstructive Lung Disease 2023 report for resource-limited settings: tracing the G in the GOLD

Bruce J. Kirenga[1,2], Patricia Alupo[1], Frederik van Gemert[3] and Rupert Jones[4]

[1]Makerere University Lung Institute, Kampala, Uganda. [2]Department of Medicine, Makerere University, Kampala, Uganda. [3]Department of Health Sciences, Groningen Research Institute for Asthma and COPD, University Medical Center Groningen, Groningen, The Netherlands. [4]Research and Knowledge Exchange, Plymouth Marjon University, Plymouth, UK.

Corresponding author: Bruce J. Kirenga (brucekirenga@yahoo.co.uk)

Shareable abstract (@ERSpublications)
COPD guidelines still have blind spots for low-resource settings. If GOLD is truly to be considered a global report on COPD, more attention will need to be paid to practical solutions in global settings. https://bit.ly/3zusjOd

Cite this article as: Kirenga BJ, Alupo P, van Gemert F, *et al*. Implication of the Global Initiative for Chronic Obstructive Lung Disease 2023 report for resource-limited settings: tracing the G in the GOLD. *Eur Respir J* 2023; 61: 2300484 [DOI: 10.1183/13993003.00484-2023].

This single-page version can be shared freely online.

Copyright ©The authors 2023.

This version is distributed under the terms of the Creative Commons Attribution Licence 4.0.

Received: 27 March 2023
Accepted: 30 March 2023

To the Editor:

The new Global Initiative for Chronic Obstructive Lung Disease (GOLD) 2023 report provides a very useful synthesis of available scientific evidence to guide COPD management, research and prevention, as always [1, 2]. Important changes include the revision of the definition of the condition and the replacement of groups C and D with E, which highlights the importance of exacerbations in COPD [2].
